# Supplementary material for: Roadside sales activities in a South Pacific Island (Bora-Bora) reveal sustainable strategies for local food supply during a pandemic
Source: PLoS One. 2023 Apr 27;18(4):e0284276. doi: 10.1371/journal.pone.0284276 (PMC10138458; doi:10.1371/journal.pone.0284276)
Supplement: Attachment — Submitted filename: Review Bora Bora .docx [file pone.0284276.s002.docx]

Review of “Roadside sales activities in a South Pacific Island (Bora-Bora) reveal sustainable strategies for local food supply during a pandemic”

Economic data tend to be based on official numbers, but informal systems can be as important, particularly in developing countries. It is important to understand informal food production and trade systems as it helps understand strengths and limitations to improve food security and resilience in the face of different disasters, including covid, but also climate change related impacts. This article contributes to building such understanding.

As the authors mention, their quantitative data is limited, but these data, added to the potential of qualitative data from interviews, can provide an interesting picture of the informal trade system of Bora-Bora island, particularly their roadside stalls. Methods need to be improved to allow readers to understand what was done and why.

Language in general is good, allowing the reader to understand what the authors intend to explain. Some sections can be improved, but unfortunately doing so on a PDF is too inefficient, so I limit my comments to some unclear sections. It would be good however, to get an English edition just before re-submission.

Introduction: Could you please provide some more context about the study? Was this part of a larger study or a stand-alone study? If it is complementary to other research you have been doing, it would be interesting to make the connections through a short description or references. I assume road stalls are only one of many options of trading/accessing food. What other informal food systems are in place (a short reference to for example importance of subsistence fishing/cropping, gifted food, bartering?). Such connections might also be relevant to include in the discussion section.

Methods: This section needs to be improved to allow readers to replicate the study if they wanted to. In particular, you mention interviews, but these are treated almost as an afterthought – it’s impossible for the reader to find out what was said by interviewees and what are the authors’ insights or opinions. Nevertheless, if done properly, interviews are an important source of information. Enough detail needs to be provided in this section about them: did you conduct interviews during all your surveys (survey meaning a sampling effort – e.g. roundtrip)? how did you know which stall holders you were going to interview or not during each survey? Did you have some criteria to decide if you were interviewing that person or not (e.g. older than 18?); how did you ensure you covered the diversity of views you wanted to cover (e.g. different types of stalls, provinces, gender, ages)? How long did the interviews last? Did you interview people more than once to understand changes? Provide a link to the interview guides, so that the reader understands how you approached your themes (did you have themes?) or just the general topic. Were interviews conducted in French? Translated? Transcribed? How did you analyse the interviews? Did you code the interviews? Deductively or inductively? If this is the first time you use qualitative data, you can get an idea of what qualitative analysis is here <https://gradcoach.com/qualitative-data-analysis-methods/>. Include also any key ethics considerations, like informed consent or if you interviewed minors, did you request parents’ authorisation?

From what I can gather, you decided to use the results from your interviews to enrich the discussion, in addition to references… Please explain why you didn’t present the interview data in the results section. In the discussion the source of information becomes unclear, as you are mixing up your views, interview results and references. So I would rather have a section within results where you present interview data. But it’s your decision, depending on your data.

Results: How many people did you interview? (from each type of stall, from each province? From each gender? Age group?). You discuss that stall holders moved towards Vaitape and Povai, and you also have GPS coordinates – can you show the location of stalls (and changes?) on your map(s)?. Interview results would be enriched if you provided a few quotes to illustrate key points. In the section where you present number of stalls, proportions and changes, try to be consistent, so that it’s easier for the reader to follow your key points, rather than having to move up and down to understand if x proportion is equivalent to the proportion mentioned before for another type of stall.

Discussion – The use of sources needs to be revised. For example, some statements are not properly referenced: e.g. “Many people in Bora-Bora have jobs related to the tourism industry and had to find an alternative source of income during the pandemic [11]”. This reference, however, is not about Bora-Bora and the main points are that the loss of tourism in dependent countries has resulted in increased poaching and extractive industries taking advantage… If there are no references in Bora-Bora of whatever idea you are proposing, but you find that it happened elsewhere, you would need to say something like “…, as has been reported elsewhere/name of place.”. Review all references to make sure that the statements correspond to the reference, and if not, clarify the relationship between the statement and the reference provided. If interview results are kept in this section, you will also need to clarify which statements are from this study (according to one/some/most of our interviewees… or provide a short quote and in brackets a code for that interviewee; e.g. female “tui” stall holder from Vaitape. If on the other hand you are proposing an idea, you can clarify to the reader that it is yours “We propose here that x happened because of y” (I am unsure of the journal politics regarding passive voice vs. direct statements, so re-write accordingly if necessary).

Study limitations – as I am not 100% sure about your sampling strategy, I don’t know why you didn’t record certain data on the stall when you interviewed people (e.g. amount of fish, crops, people manning the stall). If not included already, mention all key items to record in potential future studies, and explain why you didn’t recorded them (can be in methods if more appropriate).

Availability of data: the authors state that the data are fully available upon request, but this hasn’t been stated in the paper.

Specific comments:

An Ethics statement is missing. As the study involved human participants (interviews), this is a requirement, even if the organisation/country doesn’t require one (which is rare nowadays). The authors need to check if their organisation and/or country do have a human ethics approval system that they were not aware of; if that is the case, this step would have to be fulfilled (include approval number/code if that’s the case); if not possible, explain what steps were taken to protect the rights of participants. The minimal information to include is that explanation of the purpose of the project, benefits/risks and treatment of personal information was provided, and that all participants agreed to participate (informed consent). Include a short statement in the methods section, or wherever the journal suggests.

Row 23-25: “In many Pacific
24 Islands, people returned to nature in order to provide a much-needed source of food for the local
25 population” – it is unlikely that this was the reason for Pacific Islanders to “return to nature” – most likely they did so to provide for themselves, their families, or to generate income.

Row 42: “Small Island
42 Developing States (SIDS) are one region particularly vulnerable to global social, economic and
43 environmental crises” – SIDS is not a region – there are SIDS in different regions (e.g. the Pacific, the Caribbean… - correct sentence)

Row 48: Were exports blocked in French Polynesia? The references seem to be from other places and is presented as an important argument. If people need to turn to local resources to meet food requirements – is it because exports are blocked, or imports? Maybe you can use “trade disruptions” instead, a more general term. But please include the direct link to “increasing pressures on local resources” – is it because imported food was less available? Or maybe the real reason is because more people had income shortages, and local food is likely to be cheaper than imported food (or almost free if you fish/produce it yourself)? I suggest to re-write this sentence including a link to the Polynesian context in particular…

Row 93: Can you provide more context about the differences between the provinces? Some indications are scattered through the document, but some basic information should be provided early on, with the map, like population; economic status if available; main activities including tourism, fishing and agriculture; road quality/access…

Row 95: The map provided seems to have some holes (coloured contours don’t match up) – are these landlocked provinces? Please include the road(s) that you followed in your map, and the location of the stalls (it might be necessary to show a map for each period?). Include an inset with a general location of Bora-Bora in relation to a more general world/region map. The quality of the map could be improved.

Row 103: Semi-directive interviews? Do you mean semi-structured? The use of interviews is poorly explained both in methods and in results. If interviews were used, keep in mind that this is one of a collection of respected social-science methods (albeit mostly qualitative in nature): the design of the study and the reporting of the associated methods (sampling, implementation, analysis) needs to be included to legitimise the data collected. I suggest following a guiding document on use and report on interview results (a simple one in Young et al 2017 - A methodological guide to using and reporting on interviews in conservation science research; more in-depth: Sage publications on qualitative research in general, or interviews in particular): Key information to include (from Young et al):

Here, the following information is missing: sampling strategy (did you interview all stall holders or did you try to cover a diversity of them (e.g. the different types of stalls described, different genders, different age categories, different districts? Different sampling periods?); how many people did you interview from each criteria (at least types of stalls); did you repeat interviews with the same participants for the different periods?; did you conduct a pilot to test the interview guide?; provide access to the interview guide as supplementary material or reference to where people can find it; methods in the field – consent before starting interview? interviews were carried out at the stalls? How long did they last? Did you record them and transcribe them? How did you analyse the information from interviews? Coding strategy? Other ethical considerations: how did you ensure that the data stays anonymous and confidential.

Rows 105-118: the use of the term “Survey” is confusing here… This is more a classification of the type of stalls that you surveyed… So you probably mean groups, type of stalls, or something like that. You’ve already used survey to mean (I think, it needs clarifying as well) 1 round-trip around the island… Change the term throughout the document.

Rows 127-129: The sampling strategy needs to be clarified: it says that only new/absent stalls were identified… However, you must have had a baseline at an early point to decide that a stall was new or absent; this is not explained.

Row 130: You say “the three census periods”, but up to this point you haven’t explained what these are (other than in the abstract, which is independent). You can improve the description of all the different combinations (i.e. “period” x “survey” x “round-trip/district targeted trip” x “district” x “week/weekend day”) – A table could be easier to understand for your readers and would avoid a long text to try to put in all that info, but this is just a suggestion. E.g.:

|  | Period 1 |  | Period 2 |  | Period 3 |  |
| --- | --- | --- | --- | --- | --- | --- |
|  | Week day | Weekend day | Week day | Weekend day | Week day | Weekend day |
| Province x | # round-trips  # targeted trips | # round-trips  # targeted trips | Etc. | Etc. | Etc. | Etc. |
| Province y | # round-trips  # targeted trips | Etc. | Etc. | Etc. | Etc. | Etc. |
| Province z | # round-trips  # targeted trips | Etc. | Etc. | Etc. | Etc. | Etc. |
| Total | # round-trips  # targeted trips | Etc. | Etc. | Etc. | Etc. | Etc. |

I am assuming here that each time you went out, you recorded information on any kind of stall, and not that you had cooked meals days and ignored fish stalls those days? If that’s not the case, you would need to include that information as well…

Row 131: No need to repeat “similar to human population censuses” – this has been said and it’s not particularly important. Check for other instances in the rest of the paper for repeated/unnecessary information…

Rows 134-136: descriptive statistics can be thorough… did you mean inferential statistics? I feel you are trying to justify why your study didn’t include more difficult statistics. I believe that a descriptive study, supported by qualitative data (interviews) is valuable enough, particularly because the stalls are part of an informal food system – key for the local populations as income generators and as a contributor to food security; sometimes more important than “official” systems! Informal systems are always more difficult to track with traditional quantitative methods, and for this reason descriptive analysis and qualitative data become even more important. This doesn’t mean, however, that certain protocols can be ignored, to show to your readers that descriptive statistics and qualitative methods were designed and implemented to ensure your results are valid (see comment above about interviews). Limitations of a study are usually presented at the end of a paper, where you provide recommendations for future studies.

Row 139: …, on average 63%, most stalls… the 63% should be in brackets; however, I’d consider getting rid of this average here, saying simply that most stalls were in Vaitape… For me, the evolution of this proportion (59 to 60 to 69%) is more interesting and shows me at the same time that the proportion was high for each period and overall… Consider doing the same for the other stall types.

Row 160: The table provides information about the 4 types of stalls – put it either at the beginning of the descriptions (my preference) or at the end, rather than in the middle… If you put the table at the beginning, include a short reference to it in the text just before.

Rows 169- 171: add “respectively” to clarify that 44% and 50% refer to Anau and Matira, and clarify that it refers to the change between period 2 and 3.

Row 177: when you talk about ‘*tui*’ sellers, do you mean only people who sell fish bundles, or any fish seller? As you said before that ‘*tui*’ is “bundle”, you would need to avoid the term tui seller, or clarify in the methods that you will be referring to both types of fish sellers (bundle AND bags) as “tui sellers” (if that’s how everybody calls them, regardless of how they actually sell the fish?).

Rows 183-185: Saying that the changes were mainly in Povai is a bit misleading, as they started with only 1 stall, meaning that ANY change will be significant (1 more and that’s already 100%). Check if for the rest of the text, there are other misleading conclusions, where it might be better to talk about numbers rather than proportions…

Row 190: It’s not clear if the 55% increase refers to the overall increase (period 1 to period 3)? Try to follow the same pattern when you present the results for each stall type, so that it’s easier to the reader to understand the main results.

Row 191-193: Try not to repeat the information in the table and in the text – most people won’t be familiar with the provinces’ names, so it’s quite hard to follow what you are trying to say (i.e. 2 new here, one here and another there, then one less over there…) – this information is instead quite clear in the table.

Row 196: It’s not the distribution that changed (as long as we know!), but the number of roadside stalls.

Row 206: Did you mean direct rather than indirect? Sellers are mostly selling directly to end-consumers, aren’t they?

Row 213-215: What is the source of this information? Interviews? Reference?

Row 217: quantity or ~~frequency of~~ fish … not sure what frequency refers to? The number of times they (stall holders) were on the road each week for example? Clarify, as putting it together with quantity can be confusing, as the measurements are likely to be quite different…

Row 226-227: Maybe from your interviews, do you have more information about the sellers for whom the stalls represent supplementary income vs. main income?

Row 227-229: Check what you meant by ‘…towards ~~in~~ Vaitape…’. In addition to checking this sentence, the results presented in this paper don’t include information on shifting economic activities – are you referring to data from interviews? In that case, you need to present that. Or are you proposing that the number of stalls can be interpreted as changing economic activities (stalls are only one example of “economic activities”, but you think it can be a proxy?)? If so, explain your arguments.

Row 231: “may also be a reason” – it’s common around the world to find more sales where there’s more people, so you can get rid of the doubt in this sentence…

Row 232: What is the significance for the rest of the paragraph or the study that Matira is famous for its beaches? Explain

Row 233-235: What does “moved” mean? Do they move their stalls (and they go back and forth each day they have their stall open), or they migrated permanently (with their families?)? How do you know that products are mostly for locals (during border closures that seems obvious, but after?)? What is the source of all this information? Interviews?

Row 235: either …pandemic may have been ~~as~~ an opportunity…, or …pandemic may have been SEEN as an opportunity…

Row 236: I’d start a new paragraph here, from “tourism activity…”, to discuss what might happen in the future and discuss in more detail the importance of considering the resilience of the population in the face of future events (including climate-change related) – the response of the local population to a scarcity of jobs seems like a positive trait that would help them prepare for other major events? Check arguments presented in Ferguson et al 2022, Marine Policy 137 - Local practices and production confer resilience to rural Pacific food systems during the COVID-19 pandemic.

Row 243: While not directly related to your study because you don’t seem to have data to support that marine habitats and species were under higher pressure, studies outside Bora-Bora, around the Pacific have shown that it was the case for many places (e.g. Fergusson et al 2022). You can find further references that support diversifying food production and income activities and elaborate on this point as a separate paragraph.

Row 245-248: These two sentences would be better placed with a previous paragraph that talked about the shift of economic activities.
